# The Noncanonical Wnt5a–Ca^2+^ Pathway Mediates Mitochondrial Dysfunction in the Progression of Diabetic Nephropathy via the Mitochondrial Calcium Uniporter

**DOI:** 10.1111/jcmm.70422

**Published:** 2025-02-26

**Authors:** Yang Fei, Qunzi Zhang, Junjie Jia, Li He, Sijie Gu, Dongsheng Cheng, Wenjun Lin, Haifan Xing, Niansong Wang, Ying Fan

**Affiliations:** ^1^ Department of Nephrology Shanghai Sixth People's Hospital Affiliated to Shanghai Jiao Tong University School of Medicine Shanghai China

**Keywords:** calcium, diabetic nephropathy, levamlodipine, MCU, tubular injury, Wnt5a

## Abstract

Abnormal Wnt5a expression, mitochondrial abnormalities and calcium overload have been detected in many metabolic diseases. However, the association of Wnt5a–Ca^2+^ and mitochondrial dysfunction in diabetic nephropathy (DN) progression remains unknown. We used streptozotocin‐induced DBA2/J male mice as a DN model. The mice were treated with losartan (10 mg/kg/d*12 w) or losartan (10 mg/kg/d*12 w) + levamlodipine (5 mg/kg/d*12 w). High glucose (HG) (40 mmol/L)‐induced HK‐2 cells were used for in vitro experiments. Wnt5a and mitochondrial calcium uniporter (MCU) expression, mitochondrial dynamics, morphological changes and Ca^2+^ concentration were detected in different groups. Levamlodipine, a kind of calcium channel blocker, in combination with losartan ameliorated tubular injury and reversed mitochondrial fragmentation and dynamic dysfunction more efficiently than losartan alone in diabetic mice. Wnt5a induced Ca^2+^ uptake and aggravated mitochondrial fusion–fission disorder in HG‐stimulated HK‐2 cells. In addition, increased MCU formation was found in the mitochondria of tubular cells under HG stimulation and was upregulated by the activation of the Wnt5a–Ca^2+^ pathway. Our study showed that the Wnt5a–Ca^2+^ signalling pathway was involved in Ca^2+^ overload‐induced mitochondrial dysfunction possibly through MCU in tubular injury and DN progression. A calcium channel blocker in combination with a renin–angiotensin system inhibitor (RASi) could be a promising therapeutic strategy in DN patients.

**Abbreviations**
ACR: albumin/creatinine ratio
ATP: adenosine triphosphate
BG: blood glucose
BW: body weight
CCB: calcium channel blocker
DM: diabetes mellitus
DN: diabetic nephropathy
Drp1: dynamin‐related protein 1
ER: endoplasmic reticulum
ESRD: end‐stage renal disease
FBG: fasting blood glucose
Fis1: mitochondrial fission protein 1
GBM: glomerular basement membrane
HG: high concentration of glucose
HK‐2: human renal proximal tubular epithelial cell line
KW: kidney weight
LAML: levamlodipine
LOS: losartan
MAMs: mitochondria‐associated endoplasmic reticulum membranes
MCU: mitochondrial calcium uniporter
Mfn1/2: mitochondrial fusion protein 1/2
RASi: renin‐angiotensin system inhibitor
ROS: reactive oxygen species
SBP: systolic blood pressure
STZ: streptozotocin
TEM: transmission electron microscope
Wnt5a: wingless‐type family member 5a

## 1 Introduction

DN, which occurs in 10%–30% in patients with DM, has been widely recognised as a leading cause of ESRD in most countries and manifests as albuminuria, glomerular and tubular epithelial hypertrophy, and deterioration of renal function [1, 2, 3]. Tubular injury has been increasingly implicated in DN progression; however, the underlying mechanism remains unknown.

Recent work has revealed Wnt5a signalling as a novel inflammatory mediator closely related to obesity‐associated insulin resistance and metabolic dysfunction [4, 5]. Wnt5a plays important roles in development, cell proliferation and cell migration [6, 7]. In our previous studies, we performed RNA sequencing of kidney biopsy samples from DN patients and interestingly found that Wnt5a was obviously upregulated in advanced DN patients [8]. One of our studies also showed that Wnt5a promotes renal tubular inflammation in DN by binding to CD146 through noncanonical Wnt signalling. Furthermore, a recent study found that upregulation of Wnt5a led to enhanced mitochondrial energy production, altered cell calcium homeostasis and potentially played a specific role in the development of human ameloblastoma [9]. However, there has been limited translational work investigating the relationship of Wnt5a with mitochondrial dynamics and calcium alterations in DN models.

Increasing evidence has shown that mitochondrial abnormalities play an important role in the progression of DN [10, 11]. Mitochondria are important organelles that largely contribute to the regulation of many cellular functions [12]. Cellular homeostasis is maintained by their structural and functional reactions, which are regulated by profission proteins (Drp1 and Fis1) and profusion proteins (Mfn1/2 and OPA1) [10]. In addition, mitochondria are also responsible for maintaining intracellular Ca^2+^ homeostasis because of their Ca^2+^ buffering capacity [13, 14]. Excessive Ca^2+^ uptake into the mitochondrial matrix can lead to mitochondrial Ca^2+^ overload and trigger the mitochondrial pathway of apoptosis [15]. However, the underlying mechanisms of this damage are not fully understood.

The process of Ca^2+^ influx into mitochondria is mainly regulated by the MCU complex [14]. During ischaemia reperfusion, cytosolic Ca^2+^ accumulation causes an increase in mitochondrial Ca^2+^, which activates the matrix chaperone cyclophilin D and triggers the opening of the permeability transition pore, leading to cell death [16]. The understanding of the MCU complex has rapidly increased in various cancers. MCU‐induced mitochondrial Ca^2+^ uptake was found to promote mitochondrial biogenesis, thus contributing to colorectal cancer cell growth [17]. However, to date, the biological function of MCU in DN remains unknown.

Levamlodipine (LAML), a kind of CCB, is widely used in the treatment of hypertension and its renoprotective role remains unclear [18]. Although less effective in reducing albuminuria than RASis, CCBs have shown some beneficial effects on DN in recent clinical studies [19, 20, 21, 22]. However, we still lack adequate information on the renoprotective role of LAML and the underlying mechanism in DN patients.

In the present study, we aimed to examine the mechanistic evidence of the Wnt5a–Ca^2+^ noncanonical pathway on mitochondrial dysfunction in tubular injury in DN. We explored the possible role of MCU in the process of calcium exchange and mitochondrial dynamics changes. In addition, we sought to clarify the renoprotective effect of LAML in tubular injury, which may offer a potential therapeutic strategy for DN patients.

## 2 Materials and Methods

### 2.1 Animal Experimental Design

Six‐week‐old DBA2/J (D2) male mice were purchased from Beijing HFK Bioscience, housed under a constant 12‐h light–dark cycle and allowed free access to food and water in the SPF room. They were randomly organised into four experimental groups (*n* = 10): a control group, a STZ‐induced diabetic group, a diabetic group treated with both LAML and LOS (SHIHUIDA Pharma Group), and a diabetic group treated with LOS. The diabetic state was induced by daily intraperitoneal injections of STZ (50 mg/kg body wt; Sigma–Aldrich) for five consecutive days. FBG was measured by the One Touch Blood Glucose Monitoring System (Roche) after the mice were deprived of food for 6 h. Repeat FBG measurements were taken every 2 weeks to verify hyperglycaemia. Mice with 250 mg/dL FBG at two distinct time points were defined as having diabetes mellitus [23, 24]. Treatment with LAML or LOS was initiated 4 weeks after STZ injection. After being randomly divided into four groups, mice were assigned to receive 0.9% saline (control and STZ group), both LAML (5 mg/kg/d) and LOS (10 mg/kg/d), and LOS (10 mg/kg/d), each dissolved in 0.9% saline by daily gavage for 12 weeks. All animal procedures used in this study were approved by the Laboratory Animals Ethical Committee of Shanghai Sixth People's Hospital Affiliated to Shanghai Jiao Tong University School of Medicine (Approval number: 2021–0183).

### 2.2 Assessment of Physiological Features and Renal Functions

BW, blood glucose, systolic blood pressure (SBP) and urine collection were conducted monthly. Blood glucose was measured via a Roche Accu‐Chek Advantage Meter. The serum creatinine level was measured using the Creatinine Assay Kit (BioAssay Systems, DICT‐500) according to the manufacturer's instructions. The 24 h urine of mice was collected by using metabolic cages. Then, both urine creatinine and albumin levels were assayed by ELISA kits (Abcam, ab204537 and ab108792). The urinary ACR (in mg/g) was defined as urine albumin (mg/dL)/urine creatinine (g/dL). Systolic blood pressure was measured at room temperature by a tail cuff method using an MK2000 blood pressure monitor (ALCOTT BIOTECH CO., LTD, Shanghai, China) under conscious conditions.

### 2.3 Histology and Immunohistochemistry

Kidney sections were stained with HE and Masson solutions and imaged by light microscopy (original magnification ×400). Twenty images were randomly chosen to assess and calculate the histological injury, and all quantitation was performed by two blinded investigators.

All glomeruli in each kidney section were graded and scored by mesangial matrix index, which was quantified as the proportion in percentage of the PAS‐positive material to the total area of each glomerulus by determining the colour intensity with ImageJ software (the National Institutes of Health, Bethesda, MD, USA). The tubulointerstitial area in the cortex was observed and graded as follows: score 0: no tubular injury; score 1: < 10% of tubules as injured; score 2: 10%–25% of tubules injured; score 3: 25%–50% of tubules injured; score 4: 50%–74% of tubules injured; and score 5: > 75% of tubules injured, as previously described [25]. For IHC staining, sections were treated with diaminobenzidine or NovaRed substrate (DAKO Labs, K3468), followed by counterstaining with Mayer haematoxylin and examination. IHC staining was scored independently by two pathologists [percentage of positive cells: five categories: 0 (< 10%), 1 (10%–25%), 2 (25%–50%), 3 (50%–75%) and 4 (75%–100%); intensity: four categories (from low to high): 0, 1, 2 and 3; and a final IHC staining score (intensity score percentage score) was determined] [23]. In addition, mouse kidney specimens were further cut into ultrathin sections (120 nm) and subsequently observed under a Philips CM‐100 transmission electron microscope. Then, the mitochondrial area, circularity and cristae width were detected by ImageJ. GBM thickness was determined by the orthogonal intercept method [26].

### 2.4 Cell Culture

A conditionally immortalised human renal proximal tubular epithelial cell line (HK‐2) was used and routinely cultured in DMEM containing 10% foetal bovine serum at 37°C. Then, the cells were treated with a high concentration of glucose (HG, 40 mmol/L) for 24 h, 48 h or 72 h.

For the overexpression of both Wnt5a and MCU, the cells were transfected with pCMV3.0 cDNA plasmids (Sino Biological, HG17580‐CH, HG20767‐UT) by using Lipofectamine 3000 and P3000 reagent (RiboBio, Guangzhou, China) according to the manufacturer's instructions. For Wnt5a silence experiments, the cells were treated with siRNA (RiboBio, Guangzhou, China). Levamlodipine (5 mmol/L, SHIHUIDA Pharma Group) was used to pre‐treat the cells for 4 weeks and then added to the media alone or with HG.

### 2.5 Living Cell Image

The mitochondrial morphology was evaluated by MitoTracker Deep Red dye (Invitrogen, mp07510, USA, 644–665 nm). MitoSOX Red mitochondrial superoxide indicator (Invitrogen, mp36008, USA, 510–580 nm) is a novel fluorogenic dye for the highly selective detection of superoxide because mitochondrial superoxide is generated as a by‐product of oxidative phosphorylation. The nuclei were counterstained with Hoechst (Invitrogen, USA), and all microscopic images were recorded using a confocal microscope (Zeiss). A total of 10 fields of cells from each treatment group were randomly selected (100 cells/group).

### 2.6 Western Blotting (WB) and Immunoprecipitation (IP) Analysis

Total protein was extracted from cells and renal tissues with radioimmunoprecipitation assay (RIPA) lysis buffer (Beyotime Tech, Shanghai, China). Mitochondrial protein was extracted from the cells according to the manual of a mitochondrial isolation kit (ThermoFisher, 89874, USA). For WB analysis, the membrane was incubated overnight at 4°C with specific primary antibodies. Wnt5a and cyto‐C levels were normalised to β‐tubulin levels, while mitochondrial protein expression was normalised to mtSH70 expression. For co‐IP assessment, cultured cells were lysed in IP lysis buffer and then prewashed with protein A/G plus agarose (Santa Cruz Biotechnology). Thereafter, the cells were incubated with the appropriate antibodies overnight at 4 °C with gentle agitation. The resultant protein–antibody immunocomplexes were precipitated with protein A/G plus agarose, and these complexes were then evaluated by WB analysis.

### 2.7 Ca^2+^ Measurement

The concentration level of Ca^2+^ both in cells and mitochondria was determined by ELISA with a calcium assay kit (ab102505, Abcam, China) according to the manufacturer's instructions.

### 2.8 Statistical Analysis

The data are expressed as the means ± SEM. Comparisons between two datasets of continuous variables were performed with Student's t test, and ANOVA with Bonferroni correction was used to analyse three or more datasets. Statistical analysis was performed with SPSS (version 25.0) or GraphPad Prism (version 6.0) software. *p* < 0.05 was considered to indicate statistical significance.

## 3 Results

### 3.1 CCB Ameliorated Kidney Injury in STZ‐Induced Diabetic Mice

**FIGURE 1:**
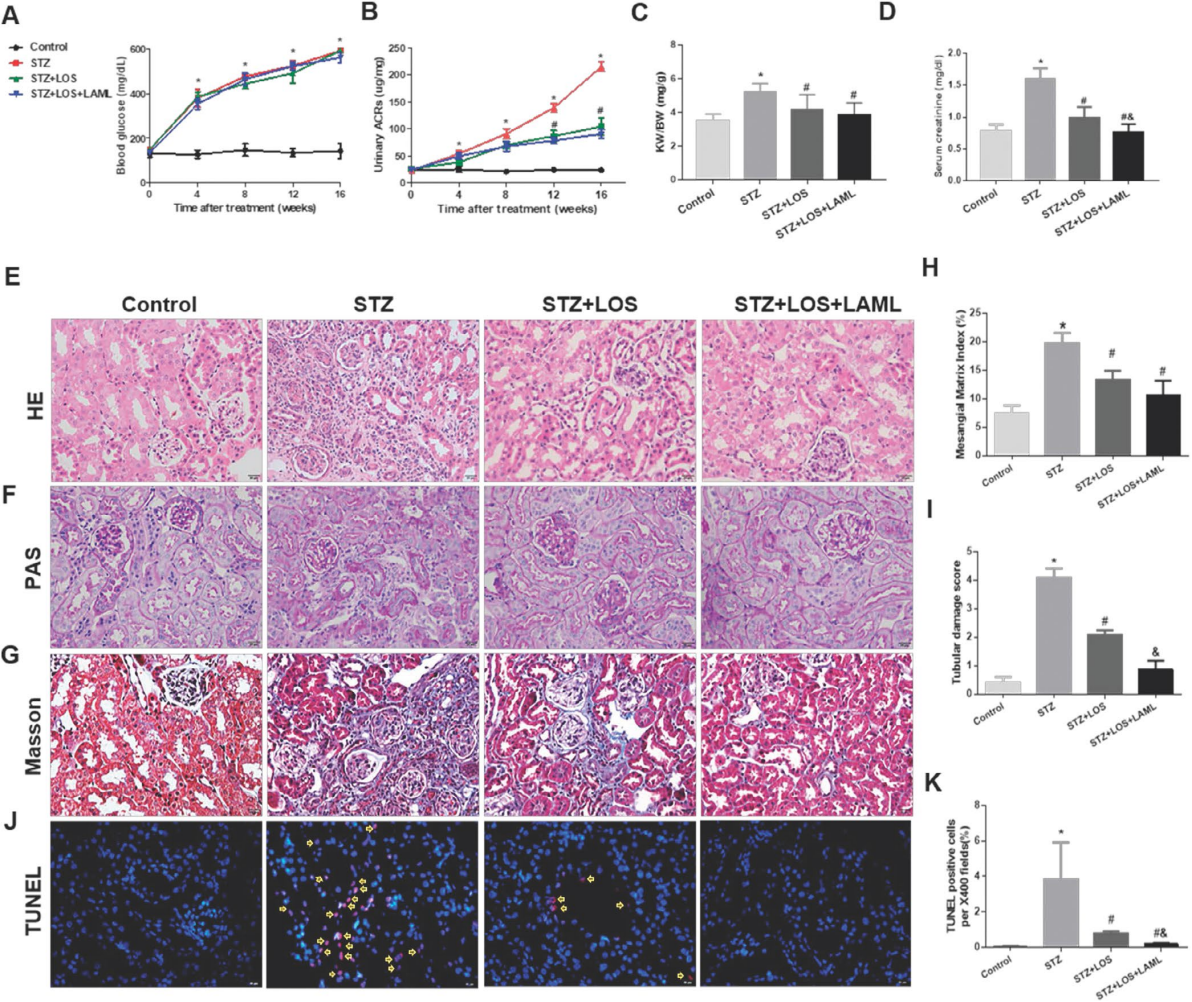
Aggravated renal tubular injury in diabetic mice was attenuated by the combination of LAML and LOS. A–D, Blood glucose levels (A), urinary ACRs (B), kidney weight to body weight (KW/BW) ratios (C) and serum creatinine levels (D) were examined in different groups. E and F, Renal histology evaluations of different groups were performed with HE staining (E) and PAS (F), and extracellular matrix accumulation and collagen fibre deposition were determined with Masson staining (G) in different groups (original magnification, 400×; bars = 20 μm). J and K, Representative photographs and quantification analysis of renal cell apoptosis in different mouse groups after TUNEL staining (original magnification, 400×; bars = 20 μm). H and I, Quantitative analyses with damage scores ranging from zero to five indicated that the combination of LAML and LOS treatment ameliorated the kidney changes after STZ induction of diabetes better than LOS alone. The results are presented as the means ± SEM. *n* = 10 for each group. *p < 0.05 compared with the control group; #*p* < 0.05 compared with the STZ group. &*p* < 0.05 compared with the STZ + LOS group. Control, nondiabetic mice with saline gavage; STZ, STZ‐treated diabetic mice with saline gavage; STZ + LOS, STZ‐induced diabetic mice with LOS administration alone; STZ + LOS + LAML, STZ‐induced diabetic mice treated with both LAML and LOS.

The STZ‐induced D2 mice showed higher blood glucose levels, increased KW/BW ratios with pronounced urinary ACRs and higher serum creatinine levels than did control mice. Compared with those treated with LOS alone, diabetic mice treated with both LAML and LOS had lower urinary ACRs, KW/BW ratios and serum creatinine levels, but no significant hypoglycaemic changes were found between this group and the control group (Figure 1A–D). There were no significant changes in SBP among the different groups (Table S1). Histologically, diabetic D2 mice had obvious renal tubular dilatation, mesangial matrix deposition and tubulointerstitial fibrosis as determined by HE and Masson staining when compared with control mice (Figure 1E–G). However, the administration of both LAML and LOS attenuated the morphological damage and tubular changes in STZ‐induced diabetic mice. The improvement was more significant than that in cells treated with LOS alone (Figure 1E–I). Furthermore, diabetic mice treated with LAML and LOS presented fewer TUNNEL‐positive cells than STZ controls and those treated only with LOS (Figure 1J,K). The data suggested that LAML in combination with LOS ameliorated DN‐induced damage and tubular cell apoptosis more efficiently than LOS alone.

### 3.2 CCB Alleviated Morphological Changes in Mitochondria and Restored Alterations in Mitochondrial Dynamics‐Associated Proteins in STZ‐Induced Diabetic Mice

**FIGURE 2:**
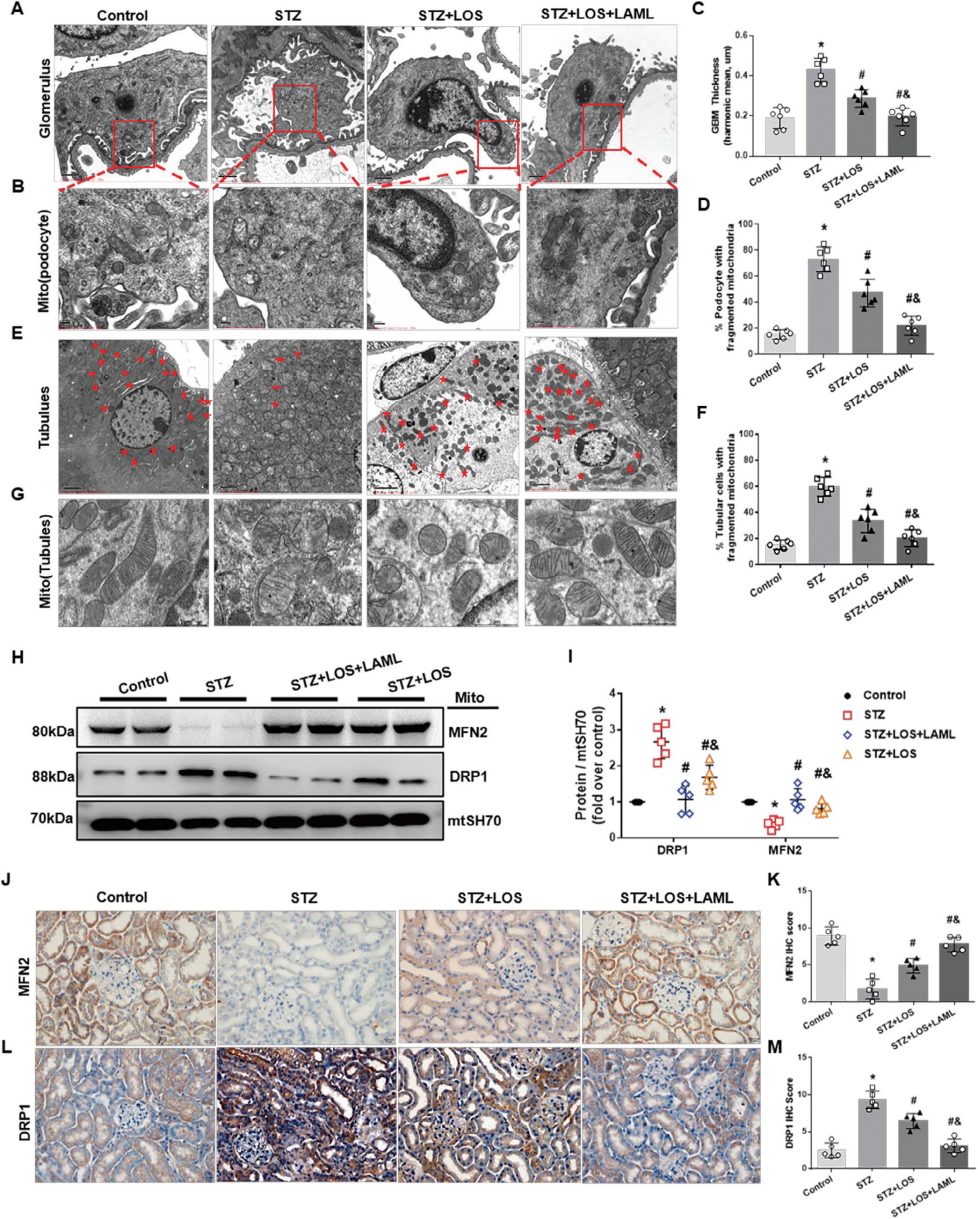
Combination of LAML and LOS alleviated mitochondrial fragmentation and restored alterations in mitochondrial dynamics‐associated proteins. A–F, Quantitative analysis and representative photographs of the fragmented mitochondria in the podocyte of the glomerulus (A, B, D) and tubules (E, G, F) in the four groups were from the TEM evaluation. C, GBM thickness in the four groups was detected by TEM. H and I, Quantitative analysis and representative photographs of Drp1 and Mfn2 expression in different groups. J–M, Representative photographs and quantitative analysis of Mfn2 (J, K) and Drp1 (L, M) by immunohistochemistry staining in different groups. TEM, transmission electron microscopy; IHC staining, original magnification 400×; bars = 20 μm. TEM of tubules, original magnification, 1500×; bars = 5 μm, *n* = 10 per group. High‐magnification TEM images of mitochondrial ultrastructure: Original magnification, 10,000×; bars = 1 μm. The results are presented as the means ± SEM. *n* = 10 for each group. *p < 0.05 compared with the control group; #*p* < 0.05 compared with the STZ group; &*p* < 0.05 compared with the STZ‐treated LOS group.

We further examined the ultrastructure of the glomerulus and tubules by TEM. Notable GBM thickening and extensive foot process effacement in the glomerulus were found in STZ‐induced mice (Figure 2C). Furthermore, the majority of mitochondria in STZ mice were rod‐shaped or spherical with disintegrated cristae, while the mitochondria in the control group exhibited elongated cylindrical shapes with organised cristae in glomeruli (Figure 2A,B). In the STZ mice, more than 50% of cells, especially podocytes and tubular cells, showed fragmented mitochondria (Figure 2D–F). However, these alterations in mitochondrial ultrastructure were more effectively alleviated by both LAML and LOS than by LOS alone (Figure 2A–G).

In addition, we examined mitochondrial dynamics‐associated proteins in this study. STZ‐induced mice showed higher Drp1 expression and lower Mfn2 expression than control mice, as indicated by WB and q‐PCR assays. However, these changes were significantly improved by the administration of both LAML and LOS (Figure 2H,I). Consistently, immunochemistry staining showed similar results (higher expression of Drp1 and lower expression of Mfn2 in kidney sections of STZ mice than in control mice at protein levels) (Figure 2J–M). These data indicated that LAML combined with LOS reversed mitochondrial fragmentation and mitochondrial dynamics dysfunction more efficiently than LOS alone in diabetic mice.

### 3.3 Inhibition of Wnt5a–Ca^2+^ Signalling by CCB Was Associated With the Suppression of MCU in Diabetic Mice

**FIGURE 3:**
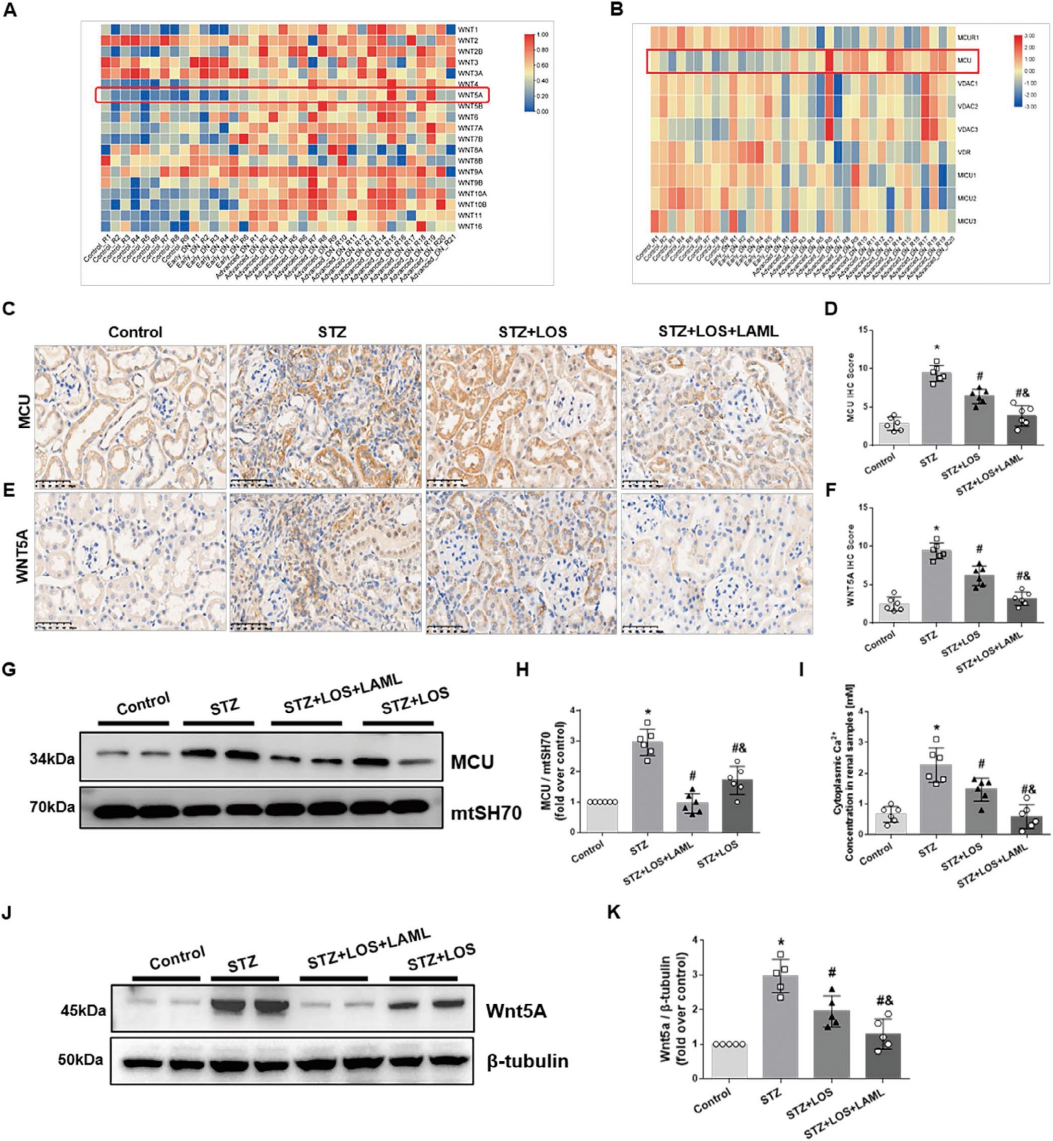
Upregulation of Wnt5a–Ca^2+^ signalling was inhibited by LAML and LOS, possibly via MCU. A, B, RNA sequencing of human kidney biopsy samples showed the expression of Wnt5a (A) and MCU (B) in DN patients. C–F, Representative photographs and quantitative analysis of MCU (C, D) and Wnt5a (E, F) by immunohistochemistry staining in different groups. G, H, J, K, Quantitative analysis and representative photographs show the expression of Wnt5a (G, H) and MCU (J, K) in different groups. I, ELISA of cytoplasmic Ca^2+^ concentrations in different groups. IHC staining, original magnification 400×; bars = 20 μm. The results are presented as the means ± SEM. *n* = 10 for each group. *p < 0.05 compared with the control group; #*p* < 0.05 compared with the STZ group; &*p* < 0.05 compared with the STZ‐treated LOS group.

In our previous study of human RNA sequences, we found that Wnt5a and MCU are closely associated with the progression of DN (Figure 3A,B) [8]. To explore the association of the Wnt5a–Ca^2+^ pathway and MCU in diabetic mice, we examined the levels of Wnt5a–Ca^2+^ pathway components and MCU in the kidney tissues of diabetic mice. Compared to the control mice, STZ mice presented an obvious increase in Wnt5a and MCU according to immunochemistry staining (Figure 3C–F). In addition, significant upregulation of Wnt5a (Figure 3J,K) and MCU (Figure 3G,H) was found at the protein level. All the changes were reversed by treatment with LAML and LOS (Figure 3C–K). Similarly, the cytoplasmic Ca^2+^ concentration was increased in the STZ mice and decreased in the group treated with LAML and LOS (Figure 3I). The data suggested that inhibition of MCU by LAML is associated with the suppression of Wnt5a–Ca^2+^ signalling in diabetic mice.

### 3.4 Wnt5a Increased Cellular Ca^2+^ and Aggravated the Dysregulation of Mitochondrial Dynamics‐Associated Proteins in HK‐2 Cells

**FIGURE 4:**
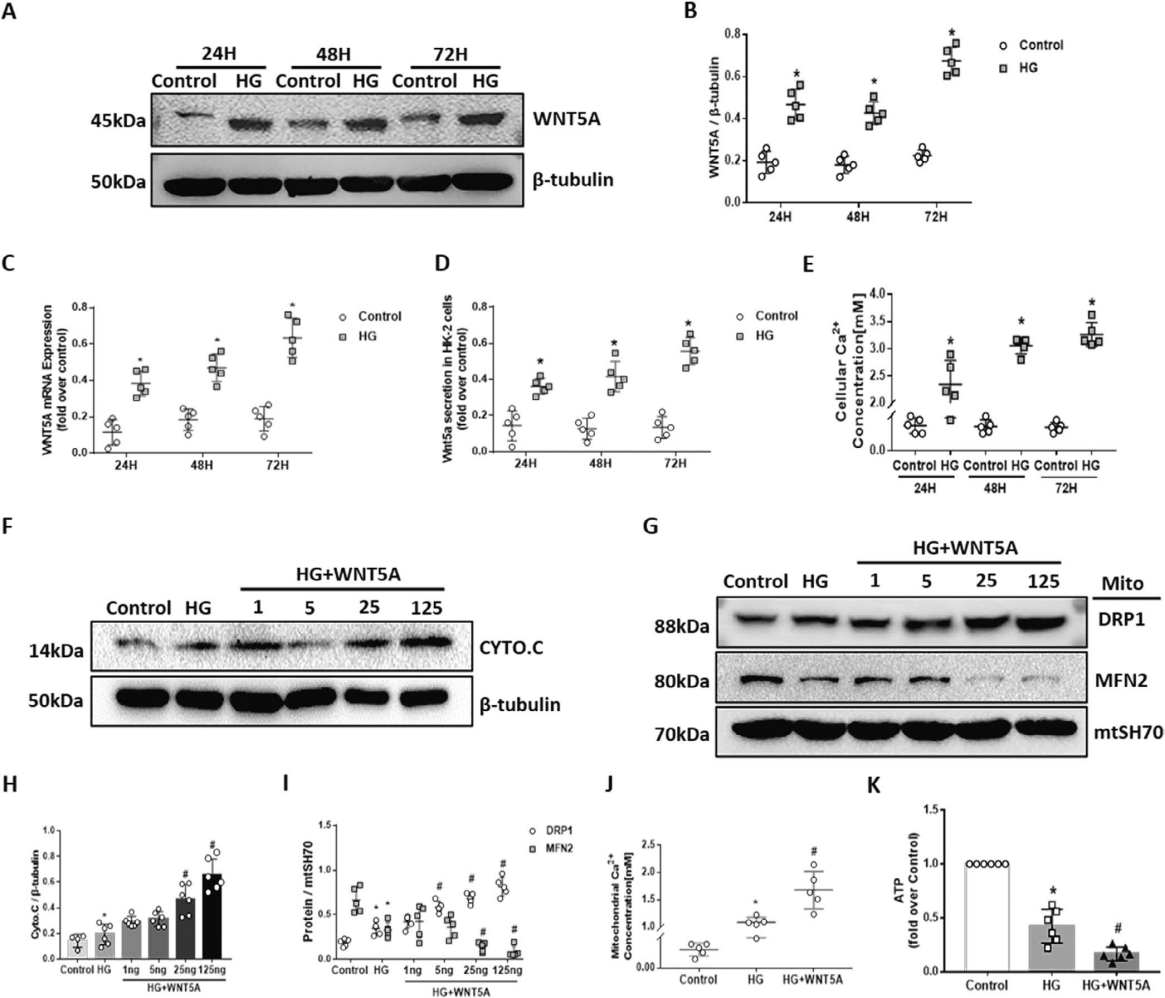
Implemented Wnt5a aggravated cellular Ca^2+^ overload and the dysregulation of mitochondrial dynamics‐associated proteins in HG‐treated HK‐2 cells. A, B, D, WB analysis and quantification analysis show the expression of Wnt5a in HK‐2 cells with different durations in HG. C, Quantitative analysis of the mRNA levels of Wnt5a in different groups. E, Quantitative analysis of Ca^2+^ concentration in different groups. F–I, Immunoblotting bands and quantitative analysis indicate the expression of cytochrome C (F, H), Drp1 and Mfn2 (G, I) in mitochondria among groups with different amounts of Wnt5a. J, ELISA analysis of mitochondrial Ca^2+^ concentrations in different groups. K, Quantitative analysis of ATP production in different groups. The results are presented as the means ± SEM. *n* = 5 for each group. *p < 0.05 compared with the control group; #*p* < 0.05 compared with the HG‐induced HK‐2 group.

To determine the underlying mechanism by which the Wnt5a–Ca^2+^ noncanonical pathway mediates mitochondrial function, we conducted an in vitro experiment. To determine the effect of HG on tubular cells, HK‐2 cells were cultured in HG medium (40 mmol/L) for 24 h, 48 h and 72 h. Compared to that in the control groups, the expression of Wnt5a increased as time was prolonged from 24 h to 72 h (Figure 4A–D). Similarly, the concentration of intracellular Ca^2+^ was increased synchronously under HG culture conditions (Figure 4E).

To further illustrate the association between Wnt5a and mitochondrial function, HK‐2 cells were treated with different amounts of Wnt5a (1 ng, 5 ng, 25 ng and 125 ng) in HG medium, mitochondria were extracted and dynamics‐associated proteins were tested in each experimental cell group. The mitochondrial cyto‐C release was obviously elevated by prolonged time interval in HG from 24 h to 72 h (Figure S1). The data showed that HG increased mitochondrial cyto‐C release (Figure 4F,H) and Drp1 expression (Figure 4G,I). Decreased Mfn2 expression (Figure 4G,I) was observed in mitochondria subjected to HG exposure with Wnt5a intervention. Consistently, a Ca^2+^ increase was observed in HG‐induced mitochondria and was more pronounced when 25 ng Wnt5a was added (Figure 4J). In addition, the loss of ATP was also detected in HG medium and was aggravated by Wnt5a (Figure 4K). The data suggested that Wnt5a–Ca^2+^ signalling is most likely involved in mitochondrial Ca^2+^ haemostasis via the impairment of mitochondrial function.

### 3.5 Overexpression of Wnt5a Accelerated Mitochondrial Dynamics Dysfunction and Ca^2+^ Overloading, Which Was Ameliorated by CCB in HG‐Treated HK‐2 Cells

**FIGURE 5:**
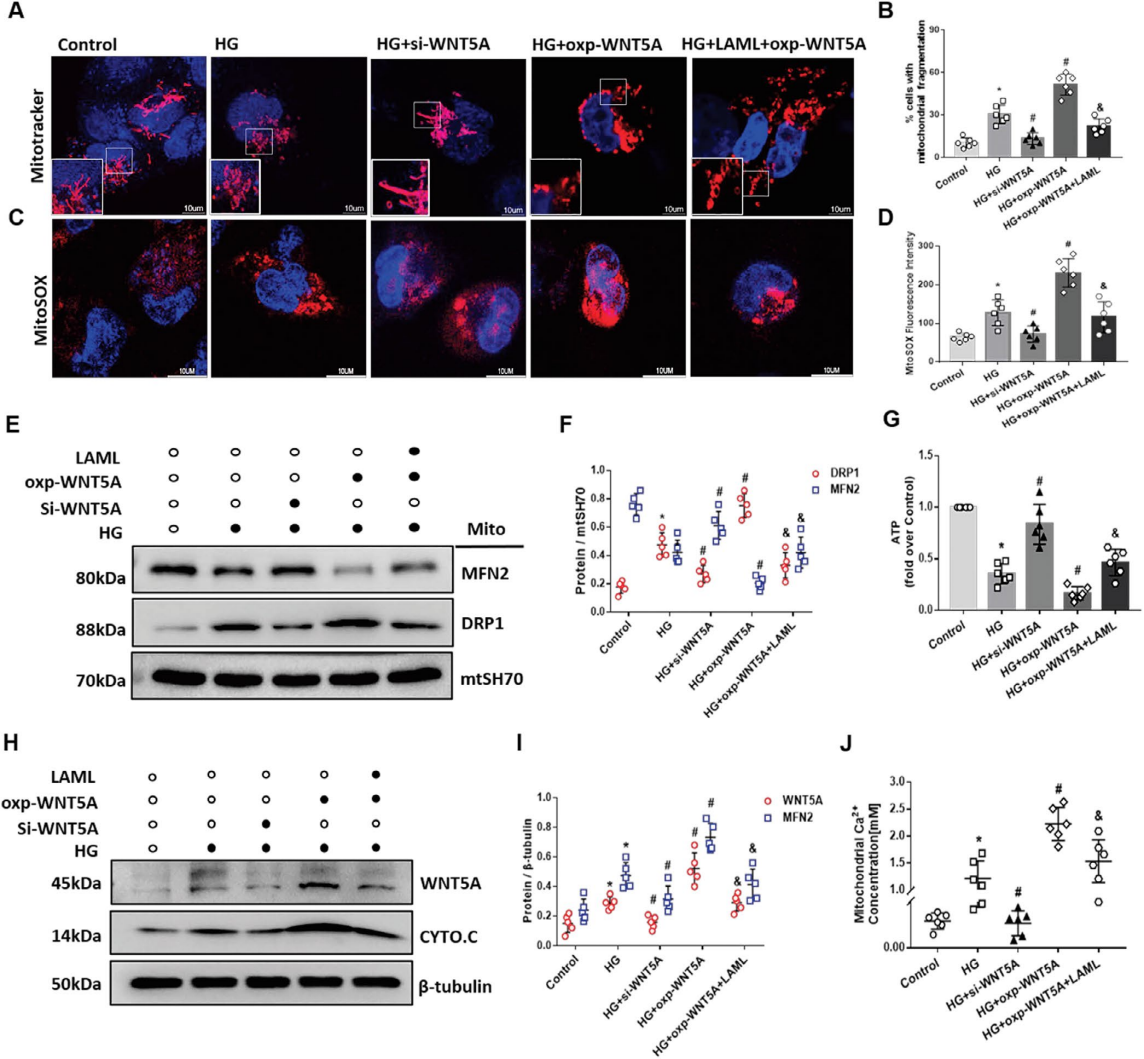
LAML inhibited the overexpression of Wnt5a and restored mitochondrial dynamics alterations, mitochondrial fragmentation and Ca^2+^ overload in HG‐treated HK‐2 cells. HG‐induced HK‐2 cells were treated with AML, Wnt5a overexpression and Wnt5a silencing. A–D, Quantification analysis and representative photographs of mitochondrial morphology (A, B), mitochondrial ROS production (C, D) confocal microscopy assessment. I, Quantitative analysis of ATP production in different groups. E, F, Quantitative analysis and representative photographs of mitochondrial Drp1 and Mfn2 in different groups. H, I, Quantitative analysis and representative photographs of Wnt5a and Cyto‐C in different groups. J, Quantitative analysis of mitochondrial Ca^2+^ concentrations in different groups. The results are presented as the means ± SEM. *n* = 5 for each group. *p < 0.05 compared with the control group; #*p* < 0.05 compared with the HG‐induced HK‐2 group.

We further examined mitochondrial fragmentation and dynamics in HG‐induced HK‐2 cells by overexpression or silencing Wnt5a with LAML intervention. MitoRed and MitoSOX were applied to detect the mitochondrial morphology and superoxide levels of the cells. The data showed that when cells with overexpression of Wnt5a were exposed to HG, almost 30% of the mitochondria transformed from their normal filamentous shape into short, rod‐like structures (Figure 5A,B). Significantly increased mitochondrial ROS production (Figure 5C,D) and ATP loss were also observed in HG‐treated HK‐2 cells overexpressing Wnt5a (Figure 5G), indicating that Wnt5a could accelerate mitochondrial dysfunction and fragmentation. However, the alterations in mitochondrial shape, ROS production and ATP loss were notably ameliorated after treatment with LAML (Figure 5A–D,G).

LAML was also found to suppress the expression of Wnt5a (Figure 5H,I) and attenuate the dysregulation of mitochondrial dynamics (Figure 5E,F). In addition, the mitochondrial Ca^2+^ concentration was significantly elevated in HK‐2 cells overexpressing Wnt5a in HG medium and was suppressed by LAML to some extent (Figure 5J). The results suggested that LAML might play a renoprotective role in mitochondria via the Wnt5a–Ca^2+^ pathway.

### 3.6 Wnt5a Increased the Expression of MCU and Mitochondrial Ca^2+^ Influx Mitochondrial Dysfunction in HG‐Treated HK‐2 Cells

**FIGURE 6:**
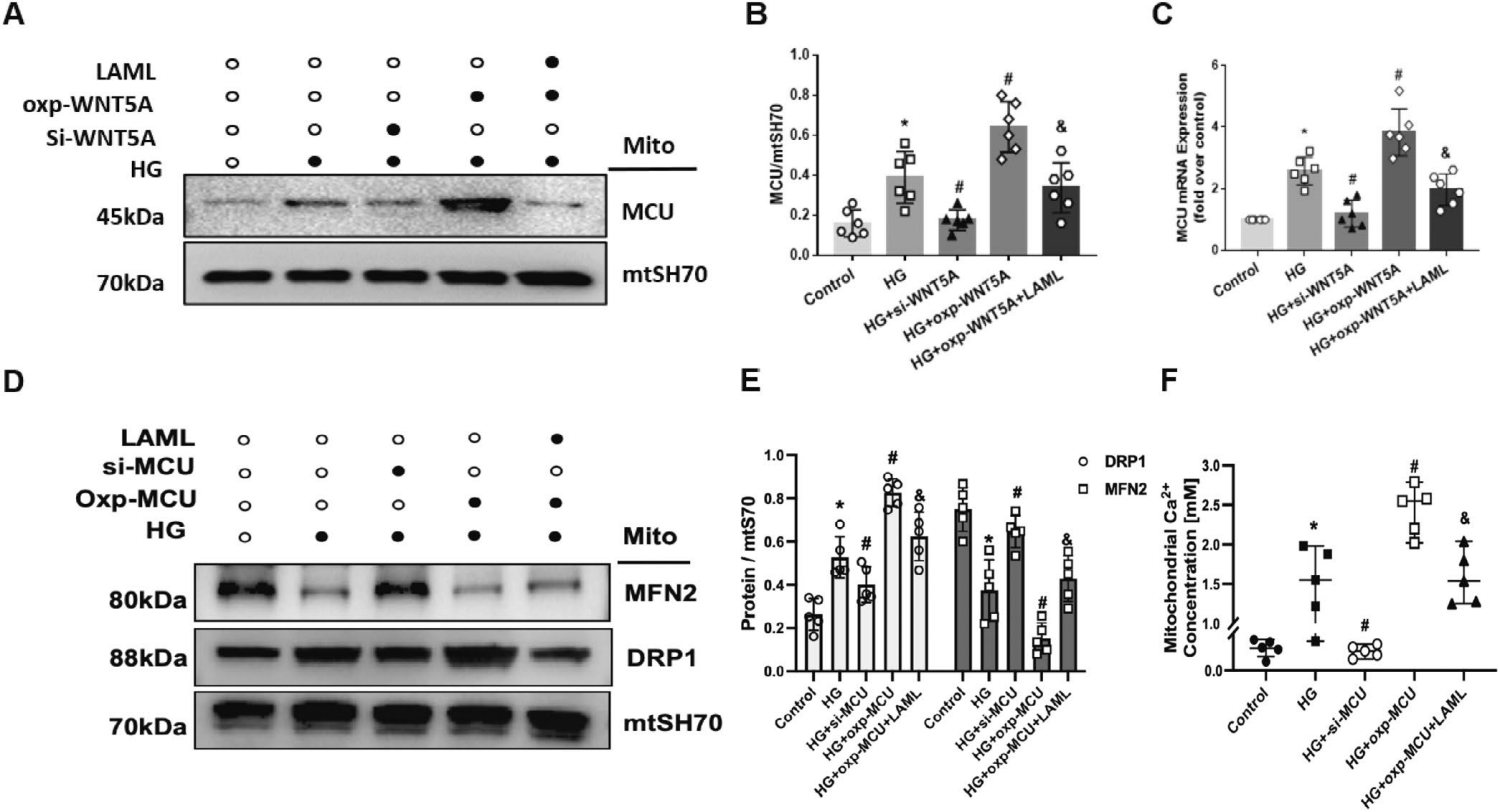
Wnt5a overexpression upregulated mitochondrial MCU and possibly led to mitochondrial dysfunction in HG‐treated HK‐2 cells. HG‐induced HK‐2 cells were subjected to LAML treatment, Wnt5a overexpression and Wnt5a silencing. A, B, Quantitative analysis and representative photographs of MCU in different groups. C, Quantitative analysis of the mRNA levels of MCU in different groups. D, E, Quantitative analysis and representative photographs of mitochondrial Drp1 and Mfn2 in HG‐induced HK‐2 cells with overexpressed MCU with or without LAML and silenced MCU. F, Quantitative analysis of mitochondrial Ca^2+^ concentrations in different groups. The results are presented as the means ± SEM. *n* = 5 for each group. *p < 0.05 compared with the control group; #*p* < 0.05 compared with the HG‐induced HK‐2 group.

Our previous RNA‐seq data showed that MCU, the core component of the complex in the electronic transport chain of mitochondria, was upregulated in the kidneys of advanced DN patients compared to those of normal controls (Figure 3B). To determine how MCU is involved in mitochondrial Ca^2+^ haemostasis, HK‐2 cells were transfected with Wnt5a under HG stimulation. Overexpression of Wnt5a significantly increased the expression of MCU at both the protein and mRNA levels in HG‐treated HK‐2 cells (Figure 6A–C). The expression of MCU was obviously decreased by LAML treatment (Figure 6A–C). Upregulation of MCU aggravated the abnormalities in mitochondrial dynamics in HK‐2 cells, as indicated by decreased Mfn2 and increased Drp1 expression in HG‐treated HK‐2 cells (Figure 6D,E). Besides, the levels of Mfn2 and Drp1 in MCU‐overexpressing HK‐2 cells were alleviated by the interference of LAML (Figure 6D). In addition, mitochondrial Ca^2+^ influx overload was observed in MCU‐overexpressing HK‐2 cells and significantly decreased by LAML compared with control cells (Figure 6F). Taken together, these findings indicate that the regulation of Wnt5a could mediate the downstream expression of MCU and thus cause the overload of mitochondrial calcium and finally lead to mitochondrial dynamics alterations.

## 4 Discussion

Diabetic tubulopathy has become increasingly recognised as an important factor related to primary renal injury in the pathogenesis of DN [27, 28, 29]. Renal tubular cells contain a large number of mitochondria to maintain cellular network balance, and increasing evidence has demonstrated that dysfunctional mitochondria play a critical role in the process of tubular injury [12, 28, 30, 31]. Recently, ROS overproduction, a decline in ATP activity, increases in mitochondrial fragmentation and alterations in mitochondrial dynamics have been shown to be closely correlated with tubular cell damage and apoptosis [27, 29, 32, 33]. Recent studies also revealed that integrated regulation of mitochondrial lifecycles in tubular cells could maintain mitochondrial homeostasis [34]. However, the underlying mechanism of these phenomena remains unclear.

To detect the impairment of tubular injury in diabetes, we chose STZ‐induced diabetic mice (DBA2/J) as the experimental model because DBA2/J mice are prone to developing DN with an early presentation of albuminuria [35]. Upregulation of the mitochondrial fission protein Drp1, downregulation of the mitochondrial fusion protein Mfn2 and increased fragmented mitochondria were detected in STZ‐induced diabetic mouse tubules. These fusion and fission events, along with the consistent results in TUNEL and staining assays of diabetic mice, illustrated that disruption of mitochondrial dynamics and mitochondrial homeostasis might lead to the mitochondrial apoptosis cascade and cell apoptosis [36].

During the experiment, we found that Wnt5a significantly increased at the protein level in diabetic kidneys but not in normal control kidneys. In vitro, we further discovered that upregulation of Wnt5a levels was obviously associated with increased Ca^2+^ levels both in cells and mitochondria, in turn, leading to damage to mitochondrial dynamics, ATP deduction, mitochondrial fragmentation and apoptosis. This might offer evidence for the role of the Wnt5a–Ca^2+^ signalling pathway in the alteration of mitochondrial dynamics and the progression of DN. Wnt proteins are now widely recognised to play essential roles in development and physiological processes by regulating vital cell functions, including proliferation, differentiation, synaptogenesis, apoptosis, cell survival, adhesion, migration, polarity, synapse formation and neuronal plasticity [37, 38, 39, 40]. Studies on neurons have demonstrated that the Wnt5a/Ca^2+^ signalling pathway regulates the mitochondrial fission –fusion process in hippocampal neurons and represents a potentially important link between impaired metabolic function and degenerative disorders [41]. Besides, the Wnt/Ca^2+^ signalling pathway has been recognised to be involved in the control and regulation of tumour development [42]. It simulates the G protein and generates messenger DAG and IP3 through phospholipase C and consequently leads to the release of Ca^2+^ in the endoplasmic reticulum into the cytoplasm [43].

This work first illustrated that the Wnt5a–Ca^2+^ signalling pathway in DN might also induce the impairment of mitochondrial dynamics in terms of fusion and fission processes and motility. The mitochondrial Ca^2+^ overload induced by Wnt‐5a and the consequent fission, fragmentation and dynamic dysfunction of those mitochondria could be physiologically relevant in tubular cells. It has been described that the traffic and movement of mitochondria are mostly modulated by Ca^2+^‐mediated changes [44]. Increased Ca^2+^ levels could lead to TCA cycle activation and oxidative phosphorylation and finally enhance mitochondrial energy production within the cell [45, 46]. On the other hand, mitochondria, as previously described, are important organelles responsible for maintaining intracellular Ca^2+^ homeostasis [47]. Considering that the endoplasmic reticulum (ER) is the main storage site for cellular Ca^2+^, ER‐related Ca^2+^ release may be dependent on Wnt‐5a/Ca2+ signalling. The increase in local mitochondrial Ca^2+^ concentration might be initiated in the ER due to the close structural interactions between the two organelles [48, 49]. Upon cellular/stress stimulation, the Ca^2+^ released from the ER‐mitochondria interface is transported through the mitochondrial intermembrane space and finally enters the mitochondrial matrix via the MCU [50]. This finding further supports that ER‐related Ca^2+^ release is dependent on downstream Wnt‐5a/Ca^2+^ signalling.

Upon cellular/stress stimulation, Ca^2+^ influx into mitochondria, which is primarily regulated by the MCU complex, acts as a pleiotropic signal that controls a broad spectrum of cellular functions [14]. Growing evidence suggests that the accumulation of Ca^2+^ in mitochondria leads to ROS production [51, 52] and that MICU1 functions as a gatekeeper to inhibit mitochondrial Ca^2+^ overload [53, 54]. A previous RNA‐seq analysis demonstrated that the increased expression of MCU in DN patients was associated with the progression of DN [8]. However, little is known about MCU‐mediated Ca^2+^ communication in the pathogenesis of DN. Only a recent study by Wei and his colleagues reported that the augmented formation of mitochondrion‐associated endoplasmic reticulum membranes (MAMs) in podocytes due to hyperglycaemic status is a critical step leading to mitochondrial calcium overload in diabetic mice [55]. With this study, we are the first to report that increased MCU formation regulated by Wnt5a–Ca^2+^ signalling aggravates the dysregulation of mitochondrial dynamics in HG‐treated HK‐2 cells. In the future, it will be interesting to assess MCU deletion as a novel treatment strategy for the progression of DN. However, further studies and additional data are warranted to demonstrate the detailed underlying mechanism by which Wnt5a–Ca^2+^ signalling regulates mitochondrial dysfunction via MCU in DN.

LAML is a traditional CCB for hypertension and other forms of cardiovascular disease. However, few studies have been conducted to determine its renoprotective role in kidney diseases, especially in DN. In our study, we confirmed that compared to LOS alone, the combination of this CCB with LOS could further enhance renal function and attenuate morphological changes in both glomerular and tubular compartments in STZ‐induced mice. The in vitro results also demonstrated that LAML could decrease cell uptake of Ca^2+^ and alleviate the alteration of mitochondrial dynamics induced by the overexpression of Wnt5a. This finding suggests that the renoprotective effect of CCB might depend on the Wnt5a–Ca^2+^ signalling pathway, which is closely correlated with mitochondrial dynamics‐induced tubular injury in diabetic conditions. In clinical practice, LOS, a kind of ARB, is well recognised and widely used in standard therapy for diabetic nephropathy. In our article, we simulated the clinical situation and conducted add‐on research of LAML and found that LAML combined with LOS had a better improvement of renal damage rather than LOS alone. It suggested that CCBs combined with RASis could be a promising treatment approach for DN patients.

There are still some limitations in the article. First, this research was just a basic experiment for demonstrating the relationship of mitochondrial dysfunction and DN progression through the Wnt5a–Ca^2+^ signalling pathway. Clinical data was essential to confirm the findings in human subjects. We also still need to further explore the calcium concentration changes, calcium channel and detect the underlying mechanism of the regulatory function of MCU in this process. Besides, the specific target of LAML, a traditional calcium channel blocker, still remains unclear.

**FIGURE 7:**
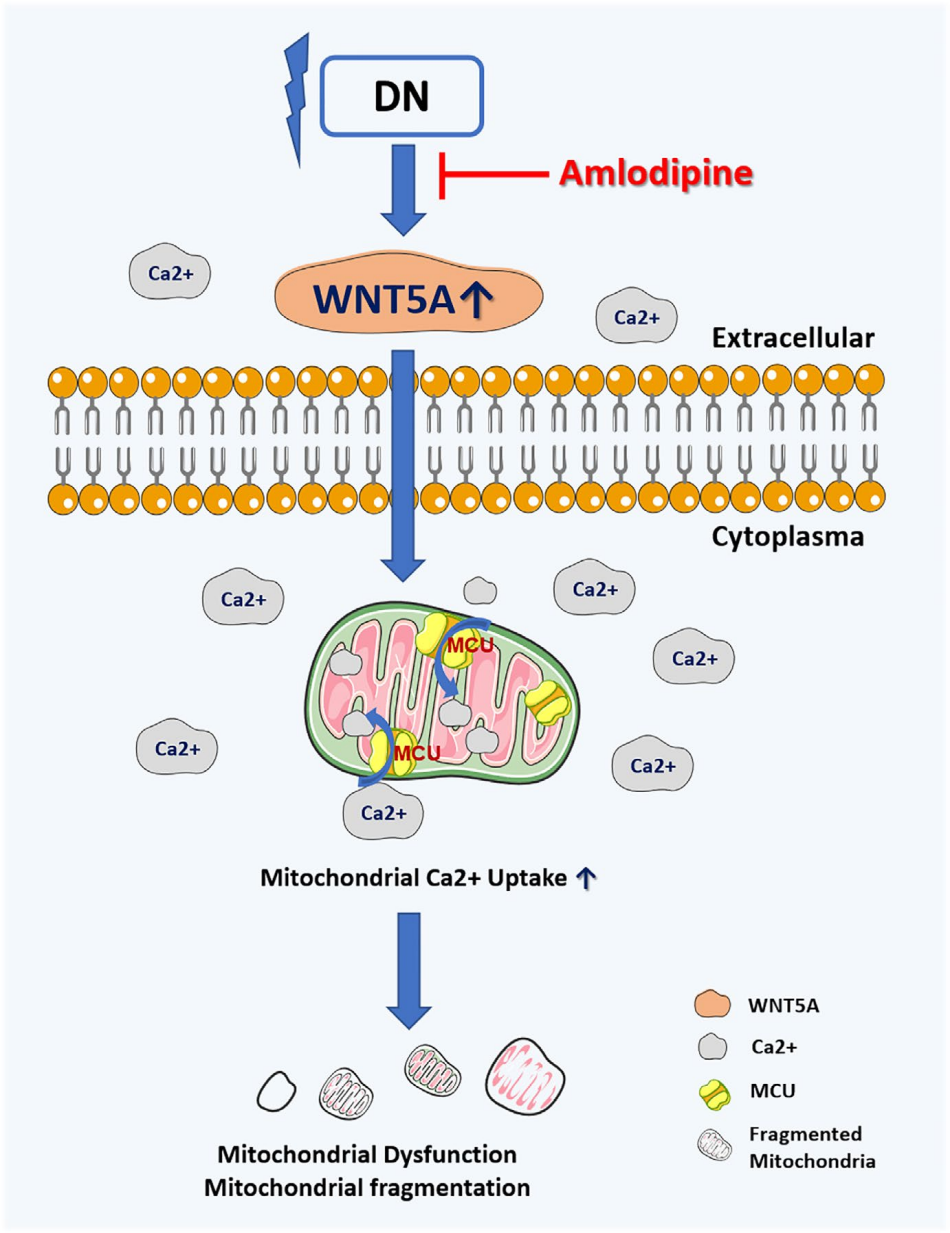
Schema of the non‐canonical Wnt5a–Ca^2+^ pathway mediating mitochondrial dysfunction in the progression of DN via MCU. Abnormities under diabetic conditions, such as hyperglycaemia, ROS or albumin overloading, can strongly enhance the expression of Wnt5a and increase the cytoplasmic Ca^2+^ concentration. MCU, as the uniporter calcium channel of mitochondria, aggravates mitochondrial Ca^2+^ overload by increasing calcium intake and finally leads to mitochondrial dysfunction and fragmentation. LAML, as a calcium channel blocker, may play a renoprotective role through the Wnt5a–Ca^2+^ pathway via MCU.

In summary, our study is the first to show that the Wnt5a–Ca^2+^ signalling pathway was involved in Ca^2+^ overload‐induced mitochondrial dysfunction in tubular injury and DN progression. (Figure 7) Dysregulation of MCU formation regulated by Wnt5a could be an important step leading to mitochondrial calcium overload, thus accelerating tubular injury in the progression of DN. Effective intervention with CCBs combined with RASis could be a promising treatment approach for DN patients.

## Author Contributions

**Yang Fei:** data curation (equal), formal analysis (equal), investigation (equal), methodology (equal), project administration (equal), writing – original draft (equal), writing – review and editing (equal). **Qunzi Zhang:** data curation (equal), formal analysis (equal), investigation (equal), methodology (equal), project administration (equal), writing – original draft (equal), writing – review and editing (equal). **Junjie Jia:** data curation (equal), investigation (equal), methodology (equal), writing – review and editing (equal). **Li He:** investigation (supporting), methodology (supporting), writing – review and editing (supporting). **Sijie Gu:** methodology (supporting), writing – review and editing (supporting). **Dongsheng Cheng:** data curation (supporting), supervision (supporting), writing – original draft (supporting), writing – review and editing (supporting). **Wenjun Lin:** investigation (supporting), methodology (supporting), supervision (supporting). **Haifan Xing:** methodology (supporting), writing – review and editing (supporting). **Niansong Wang:** conceptualization (equal), project administration (equal), supervision (equal), validation (equal). **Ying Fan:** conceptualization (lead), project administration (lead), supervision (lead).

## Conflicts of Interest

The authors declare no conflicts of interest.

## Supporting information


Appendix S1.

## Data Availability

Data available on request from the authors.
