# Supplementary material for: The Noncanonical Wnt5a–Ca2+ Pathway Mediates Mitochondrial Dysfunction in the Progression of Diabetic Nephropathy via the Mitochondrial Calcium Uniporter
Source: J Cell Mol Med. 2025 Feb 26;29(4):e70422. doi: 10.1111/jcmm.70422 (PMC11862900; doi:10.1111/jcmm.70422)
Supplement: Supplementary file 1 — Appendix S1. [file JCMM-29-e70422-s001.docx]

**Figure S1**


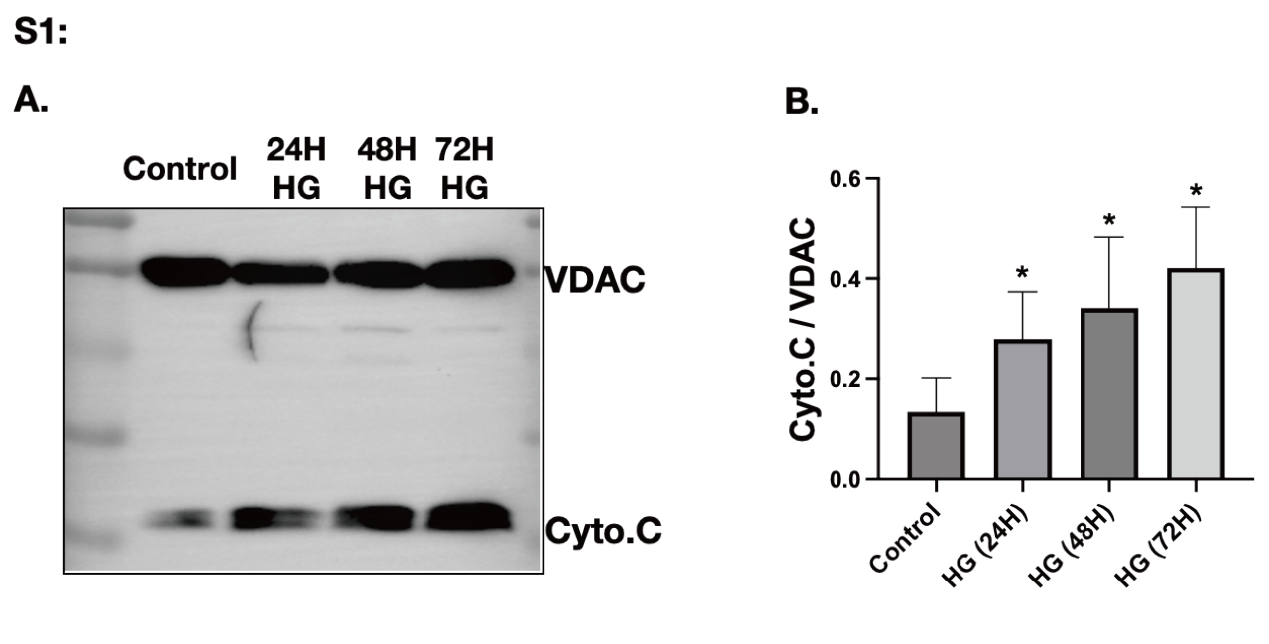


**Supplementary Figure 1. Quantitative analysis on the expression of Cytochrome C in different time interval.** *P < 0.05 compared with Control.

**Supplementary Table S1. Serial measurement of systolic blood pressure**

| **Time after**  **treatment** | **Control** | **STZ** | **STZ+LOS** | **STZ+LOS**  **+LAML** | **P Value** |
| --- | --- | --- | --- | --- | --- |
| 0 week | 132.9 ± 3.49 | 134.4 ± 3.12 | 134.4 ± 1.61 | 132.8 ± 0.26 | 0.601 |
| 4 weeks | 135.7 ± 2.47 | 136.3 ± 2.28 | 135.4 ± 1.24 | 133.5 ± 2.53 | 0.260 |
| 8 weeks | 133.0 ± 2.18 | 137.2 ± 4.44 | 134.6 ± 0.86 | 134.4 ± 1.35 | 0.119 |
| 12 weeks | 135.7 ± 3.44 | 136.7 ± 2.07 | 133.4 ± 4.52 | 132.5 ± 3.16 | 0.216 |

The data of systolic blood pressure (mmHg) are presented as the mean ± SD. One-way ANOVA was used for statistical analysis.
